# Urban Water Consumption Patterns in an Adult Population in Wuxi, China: A Regression Tree Analysis

**DOI:** 10.3390/ijerph17092983

**Published:** 2020-04-25

**Authors:** Hao Zheng, Weijie Zhou, Lan Zhang, Xiaobo Li, Jian Cheng, Zhen Ding, Yan Xu, Wenbiao Hu

**Affiliations:** 1 Department of Environmental Health, Jiangsu Provincial Center for Disease Control and Prevention, Nanjing 210009, China; zhenghao@jscdc.cn (H.Z.); hfdz@jscdc.cn (Z.D.); 2 Department of Environmental Health, Wuxi Center for Disease Control and Prevention, Wuxi 214023, China; wxcdczwj@163.com; 3 National Institute of Environmental Health, Chinese Center for Disease Control and Prevention, Beijing 100050, China; zhanglan@nieh.chinacdc.cn; 4 School of Public Health, Southeast University, Nanjing 210009, China; 101011116@seu.edu.cn; 5 School of Public Health and Social Work, Institute of Health and Biomedical Innovation, Queensland University of Technology, Brisbane, Queensland 4059, Australia; j25.cheng@qut.edu.au

**Keywords:** exposure assessment, water intake, water consumption, adults, CART

## Abstract

Understanding water intake variation is crucial for assessing human exposure to water pollutants. The correlation between water intake and demographic factors warrants further exploration. A cross-sectional study was conducted to estimate urban water consumption and its associated factors among adults in Wuxi, China, in 2015. The water consumption information was obtained by a 24-h self-report diary over seven consecutive days. A classification and regression tree (CART) analysis was applied to detect how water consumption varied with the demographic variables. Finally, a total of 1188 adults (18–87 years old) were included. The results demonstrated that the median water consumption of the adults was 1525 mL/day in summer and 1217 mL/day in winter. The results of the CART analysis demonstrated that body mass index (BMI) and age were the leading factors that were associated with water consumption in summer and winter, respectively. The water consumption threshold of BMI for men differed from women (23 kg/m^2^ vs. 18 kg/m^2^) in summer, and the threshold of age for men was also different from women (43 years vs. 21 years) in winter. In conclusion, the findings are useful for accurately assessing human exposure to water pollutants and identifying the high-risk subgroups.

## 1. Introduction

Water intake rate is a crucial parameter for the assessment of human exposure to water pollutants. When assessing the health risk of water chemicals, the calculation for the human exposure dose is a necessary step [1]. The exposure dose of chemicals depends on the water intake rate among the exposed population. A high water intake rate associates with a high-level human exposure dose when populations are exposed to the same concentration of water chemicals. 

Water that is ingested directly and water that is added to foods and beverages during preparation are the main sources of water intake for exposure assessment [2]. Estimates about water consumption patterns have been reported in the United States (US) [3,4,5], Canada [6,7], Sweden [8], and Korea [9]. In China, two large-scale studies that focus on fluid intake have been conducted [10,11]. However, food moisture was not considered in these two studies. Zhang et al. [12] estimated the total water intake among young adults aged 18–23 years, and commercial beverages that were not related to exposure assessment were also included. Estimates of water ingestion regarding exposure assessment are limited in China.

It has been demonstrated that the water intake rate is influenced by demographic, physiological, and social-economic factors [6,7,8,9,13,14]. The study conducted in four Chinese cities showed that men had a higher fluid intake than women [11]. Zhang et al. [10] suggested that socio-economic status may affect water intake in China. Wang et al. [15] reported that obese adolescents consumed more water than normal-weight counterparts in Shanghai, China. However, internationally, a default water ingestion rate of 2 L/day that was derived from large-scale nutrition surveys conducted in the US population is suggested by the Environmental Protection Agency, the World Health Organization, and other agencies [16]. The default water ingestion rate (2 L/day), as an essential exposure parameter, has been widely used in the human exposure assessment of water pollutants that may lead to uncertainties for the assessment results. To accurately estimate the water intake rate, it is preferable to establish regional water ingestion exposure values [3]. 

Several assessment methods (e.g., 24-h recall, 3-d record, or 7-d record) for collecting water consumption information have been reported in previous studies [17]. Among these studies, a 7-d self-report record was most widely used in the literature [12,18,19,20,21]. For instance, in China, two large-scale studies assessing the water intake applied the 7-d record method [10,11]. This method has been validated as an effective and reliable method by assessing accuracy [22,23]. 

In previous studies, a regression model was generally used to explore the association of water consumption with many variables, such as gender, age, and seasons [6,7,9]. However, it remains difficult to identify the threshold and high-order combination relationship of variables by this traditional model. Proposed by Breiman et al. in 1984 [24], classification and regression tree (CART) is attracting increasing interests in public health policymaking [25,26,27]. CART is a machine-learning algorithm that divides the data into subsets according to a threshold. The CART model has the ability to identify the threshold and exploring the non-linear association among risk factors [28]. Moreover, the CART model provides a non-parametric approach that can potentially better accommodate these complex interactions since they avoid some of the assumptions associated with linear regression (e.g., normality and multicollinearity) [29]. As a supplement to traditional regression models, in the present study, we used both CART and regression models to examine the association between water consumption and a variety of determinants. The combination of the two statistical methods enables us to better identify the subgroups that most likely to have a high level of water intake rate, and consequently, to identify the high-risk subgroups for the exposure assessment of water pollutants.

This paper reports a cross-sectional community-based study that was conducted among adults in urban areas of Wuxi city, eastern China. The study aims to obtain a quantitative estimate of the urban water consumption by collecting the 7-d self-report diaries of participants to explore the correlation between water consumption and demographic variables of the adults. 

## 2. Materials and Methods 

### 2.1. Study Area and Sample

This cross-sectional study was performed in urban areas of Wuxi city. Wuxi is a modernized city located in the southeast of Jiangsu Province in China, with an urban population of 3.3 million. Its average temperature is 28 °C in summer and 5 °C in winter. The gross domestic product of Wuxi was 850 billion RMB (125 billion USD) in 2015 [30].

**Figure 1:**
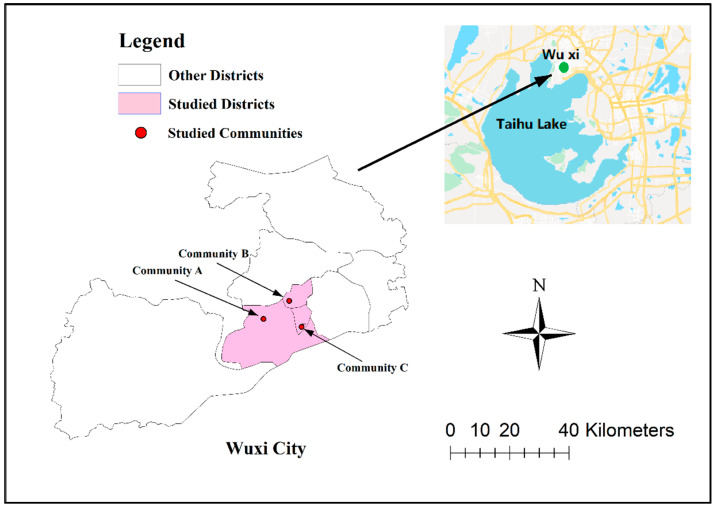
The study area with the A, B, and C communities highlighted.

A multiple-stage stratified random sampling method [31] was used to sample adults from the urban population of Wuxi city. First, three districts (Districts H, T, and N) with the same water source of Taihu Lake were randomly selected from the seven districts of Wuxi. Second, one subdistrict was selected from each of the selected three districts. Third, one community was randomly selected from each of the three selected subdistricts (Communities A, B, and C ). Finally, in each selected community, 200 healthy adults were randomly selected from all the adults registered in the member list of the community. Therefore, a total of 600 adults from three communities that were representative of the urban population of Wuxi were enrolled for the study. The study area with the selected districts and communities are presented in Figure 1. Details on the sampling method are summarized in Figure S1.

Healthy adults aged ≥18 years old were included. Adults were excluded if they were diagnosed with liver, kidney, or heart diseases that may affect water consumption.

The survey was conducted in August (summer) and December (winter) of 2015. The same subjects were surveyed in both summer and winter, if possible. If not, additional subjects with equal representation were enrolled in winter. In total, 1200 participants (person-time) were recruited in the study.

### 2.2. Data Collection

Field surveys were conducted among households to obtain data. The survey consisted of two parts: an interview and a 24-h self-report diary over seven consecutive days. All the interviewers participated in a training workshop that was held by the research group prior to the survey to learn the purposes of the study and interview techniques. All participants of this study had been informed of the aim of the survey and signed a consent form prior to the household survey. In the interview part, the interviewers collected information on the demographic factors of the adults that were hypothesized to affect the water ingestion. In the self-report diary part, each participant was provided a 24-h self-report diary in which to record the type and amount of their water consumption over seven consecutive days (starting from the day of the interview). Details on the questionnaire are provided in Table S1. 

The interviewers approached the household survey during weekends to recruit as many participants as possible. A graduated container (100 mL) was provided to each participant for accurately measuring their water consumption. The investigators monitored the diaries by telephone as a reminder during the self-report part. At the end of the survey, the investigators evaluated the completeness of all the diaries. Incomplete or no-response diaries were excluded from the final analyses.

The study protocol and all the documents were approved by the Ethics Committee of National Institute of Environmental Health, the Chinese Center for Disease Control and Prevention. All the methods in the present study were carried out in accordance with the Declaration of Helsinki. All participants provided written, informed consent for participation in the study before the interview.

### 2.3. Variables

The total water consumption was calculated as the sum of the following two sources: (a) direct water, which was defined as water that was ingested directly, including tap water, purified water, bottled water, and tea water, and (b) indirect water, which was defined as water that is added for preparation in foods, such as soup, porridge, and staple food (rice, steamed bread, noodle, rice noodle, and rice flour). Commercial beverages were not taken into account [32]. To calculate the amount of water from staple food, the median values of the water contents of staple food were used [33]. The water contents that were used to calculate the indirect water consumption in the study are listed in Table S2.

Height was measured with steel tapes (DL3796, Deli, Zhejiang, China), and weight was measured with portable scales (EB9005L, Xiangshan, Guangdong, China) by investigators. We measured height and weight in summer and winter, respectively. The body mass index (BMI) was calculated according to the equation of weight/height squared (kg/m^2^) and classified into four levels: <18.5 kg/m^2^ (underweight), 18.5–23.9 kg/m^2^ (normal weight), 24–27.9 kg/m^2^ (overweight), and ≥28 kg/m^2^ (obesity) [34]. 

We classified the participants who were mainly engaged in physical activity (blue-collar) as labor workers and the rest as nonlabor workers.

### 2.4. Statistical Analyses

#### 2.4.1. Descriptive Analysis 

Descriptive analysis was used to describe water consumption according to different seasons and demographic variables. All the data were tested for normality or for homogeneity of variances. The 25th, 50^th^, and 75th percentiles of the total water, direct water, and indirect water consumption during the seven-day period were calculated. Because most of the adults were nonlabor workers with Han ethnicity, these two variables were not included in the following analysis. 

#### 2.4.2. Univariate and Multivariate Analysis

Due to non-normal distribution of the data, univariate analysis of the water consumption (total, direct, and indirect water) and variables (gender, age, location, and BMI) was performed with the non-parametric tests (Kruskal–Wallis H test for multiple comparisons, Mann–Whitney U-test for pairwise comparisons, and Wilcoxon signed ranks est for repeated samples) [35]. Factors with *p* < 0.1 in the non-parametric tests were chosen in the multivariate analysis.

Regression analysis can be used to explore the correlations between one dependent variable and more than two independent variables. In the present study, daily water consumption is included as a dependent variable, and the demographic variables were included as independent variables. Because the data of daily water consumption was skewed in distribution, a lognormal transformation was applied before the calculation. Multiple linear regression analysis (stepwise) [36] was used to estimate how the demographic variables could affect water consumption. In the regression model, age and BMI were included as continuous variables. Dummy variables were created for location variables.

#### 2.4.3. CART Analysis

CART is considered to be a non-linear and non-parametric method based on the recursive splitting the dataset into subsets according to a certain criterion. The decision trees develop the tree model into two stages: tree building and pruning. Tree building starts with a parent node containing the entire samples. The parent node splits into two child nodes by examining each independent variable based on the impurity criterion. Each parent node in the decision tree recursively branches into two child nodes, which successively may become parent nodes forming additional child nodes. Briefly, the split that features the largest difference between the impurity of the parent node and a weighted average of the impurity of the two child nodes is identified based on the impurity criterion. The subsequent stage is tree pruning. In this stage, an optimal sub-tree of the maximal tree is identified, and the maximal tree is reduced by cutting off its overfitted parts appropriately. Both tree building and pruning continue until statistical analysis indicates that the decision trees fit without overfitting the contained dataset [29]. 

In our study, the CART model was developed to further assess the interaction effects of demographic variables on water consumption, and to identify the threshold values through partitioning the identified variables into subsets. We used the daily water consumption as the dependent variable and demographic variables (age, location, and BMI) as the independent variables. Age and BMI were included as continuous variables in the CART model. Cross-validation was conducted to select the best tree size by detecting prediction errors. The best model was defined as characterizing the smallest tree size as well as an estimated error rate within one standard error of the minimum [29]. The probability (expressed as a percentage) and mean (95% confidence interval (CI)) value of daily water consumption were used to represent the interaction effects on water consumption by variables [28]. The CART model was utilized to analyze the data in summer and winter by sex, respectively.

#### 2.4.4. Comparisons of CART and Regression Models

Relative to the regression model, CART has a number of strengths. First, CART can deal with non-normal distribution data that need to be transformed in the regression model. Second, CART can identify the high-order interaction effects and thresholds of the predictors associated with the dependent variable. Third, the predictors are ranked according to their importance in the decision trees (i.e., the first identified variable is defined as the most important predictor related to the dependent variable). Therefore, the decision trees are relatively easy to interpret for health policymakers.

All the statistical analyses were performed using R software (version 3.4.0), with the “rpart” package to conduct CART analysis. A p-value of p ≤ 0.05 was considered as statistical significance. All data were evaluated in terms of completeness and accuracy prior to the analyses.

## 3. Results

### 3.1. General Characteristics of the Participants

Among the 1200 participants recruited, 1% (12/1200) did not meet the criteria. Therefore, a total of 1188 participants who resided in Communities A, B, and C were included in the present study. During the household survey, the average outdoor temperature was 28 °C in summer and 5 °C in winter, with humidity levels of 72% and 45%, respectively. 

In summer, 50.2% (596/1188) of the adults were surveyed, and the remainder were recruited in winter. Among the participants, 48.2% (573/1188) were men and 51.8% (615/1188) were women. The sex ratio of males/females was 0.93. 

All the adults aged between 18 and 87 years, with an average age of 45 years (standard deviation of 17.1 years), while the mean (std) age was 45.4 (17.2) years for men and 45.2 (17.1) years for women, respectively. Participants were divided into three sub-age groups: 18–34 years (33.3%, 396/1188), 35–55 years (32.4%, 385/1188), and ≥55 years (34.3%, 407/1188). Most of the participants were defined as nonlabor workers (87.5%, 1039/1188) and claimed Han ethnicity (99.2%, 1178/1188). More than half of the subjects had a BMI between 18.5 and 23.9 kg/m^2^ (60.9%, 724/1188). The general characteristics of the participants are presented in Table 1.

### 3.2. The Total Water, Direct Water and Indirect Water Consumptions of the Participants

The median value of water consumption of the participants was 1351 (P25–P75: 1013–1780) mL/day. Direct water consumption accounted for 70.3% of the total water consumption. Only 15.6% (185/1188) of the participants had a water intake of >2 L/day. 

A total of 580 same individuals have been recruited in the survey in both summer and winter. The water consumption of adults with repeated measures in summer was significantly higher water than those in winter (1542 mL/day vs. 1218 mL/day, Z = −10.943, *p* < 0.001). According to gender, age groups, and location of adults with repeated measures, we also observed a significantly higher water consumption in summer than those in winter. Water consumption of the adults with repeated measures is summarized in Table S3.

Table 2 shows the water consumption of the adults according to demographic variables in summer and winter. The results demonstrated that the median water consumption of the adults was 1525 mL/day in summer and 1217 mL/day in winter. In general, significant differences in the total water consumption were identified between gender, age, and BMI in both summer and winter (*p* < 0.05), and the differences were also observed for direct and indirect water consumption (*p* < 0.05). No significant differences in total water consumption were observed between locations in both summer and winter.

### 3.3. Impacts of Demographic Variables on the Water Consumption According to Multivariate Analysis

Table 3 presents the impacts of demographic variables on the water consumption of the adults in summer and winter in multivariate analysis. The gender, age, location, and BMI were chosen as independent variables in the multivariate analysis. The results demonstrated that men consumed more water than women either in summer (95% CI: 0.099–0.273) or in winter (95% CI: 0.051–0.204). Moreover, in summer, water consumption increased with BMI (95% CI: 0.005–0.034), and a statistical association with water consumption was observed between locations. However, in winter, water consumption increased with age (95% CI: 0.002–0.006).

### 3.4. Impacts of Age, BMI, and Location on Water Consumption According to CART Analysis

The results of multivariate analysis suggested differentiated associations of water consumption and demographic variables in different seasons. To further analyze the data, a CART model was employed to explore the relationships between water consumption and age, location, and BMI for men and women in different seasons, respectively.

**Figure 2:**
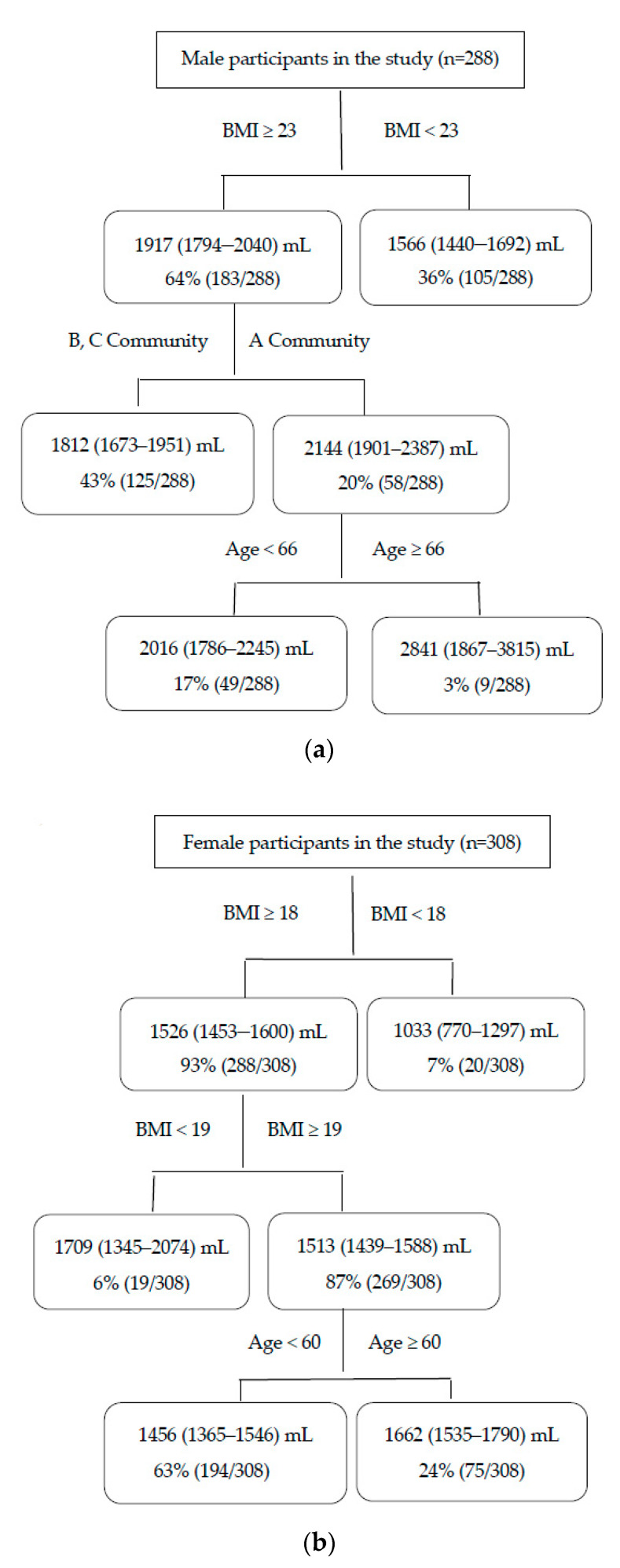
Impacts of age, BMI, and location on daily water consumption among the adults in summer according to CART analysis in the study (age: years, BMI: kg/m^2^). The figures in the rectangle indicate the mean (95% CI) values of daily water consumption and the probability; (**a**): male, (**b**): female.

Figure 2 illustrates the impacts of age, BMI, and location on daily water consumption among the adults in summer. The numbers of adults in summer were 288 for men and 308 for women, respectively. The mean (95% CI) value of daily water consumption was 1789 (1697–1882) mL for men and 1494 (1423–1566) mL for women in summer.

Figure 2a shows that for the first classification factor, men with a BMI of ≥23 kg/m^2^ (probability: 64%, mean (95% CI): 1917 (1794–2040) mL) consumed more water than those with a BMI of <23 kg/m^2^ (probability: 36%, mean (95% CI): 1566 (1440–1692) mL). Moreover, for men with a BMI of ≥23 kg/m^2^, the water consumption was higher among those who were residing in Community A (probability: 20%, mean (95% CI): 2144 (1901–2387) mL) than those who were residing in Communities B and C (probability: 43%, mean (95% CI): 1812 (1673–1951) ml). In addition, for men who had a BMI of ≥23 kg/m^2^ and were living in Community A, the water consumption at age ≥66 years (probability: 3%, mean (95% CI): 2841 (1867–3815) mL) was higher than at age <66 years (probability: 17%, mean (95% CI): 2016 (1786–2245) mL). 

For women, a BMI of ≥18 kg/m^2^ (probability: 93%, mean (95% CI): 1526 (1453–1600) mL) was detected as the first classification factor that is associated with water consumption in summer. Furthermore, women with a BMI of ≥18 and <19 kg/m^2^ (probability: 6%, mean (95% CI): 1709 (1345–2074) mL) consumed more water than those with a BMI of ≥19 kg/m^2^ (probability: 87%, mean (95% CI): 1513 (1439–1588) mL). In addition, for women with a BMI of ≥19 kg/m^2^, individuals aged ≥60 years (probability: 24%, mean (95% CI): 1662 (1535–1790) mL) had a higher water consumption than those aged <60 years (probability: 63%, mean (95% CI): 1456 (1365–1546) mL) (Figure 2b).

**Figure 3:**
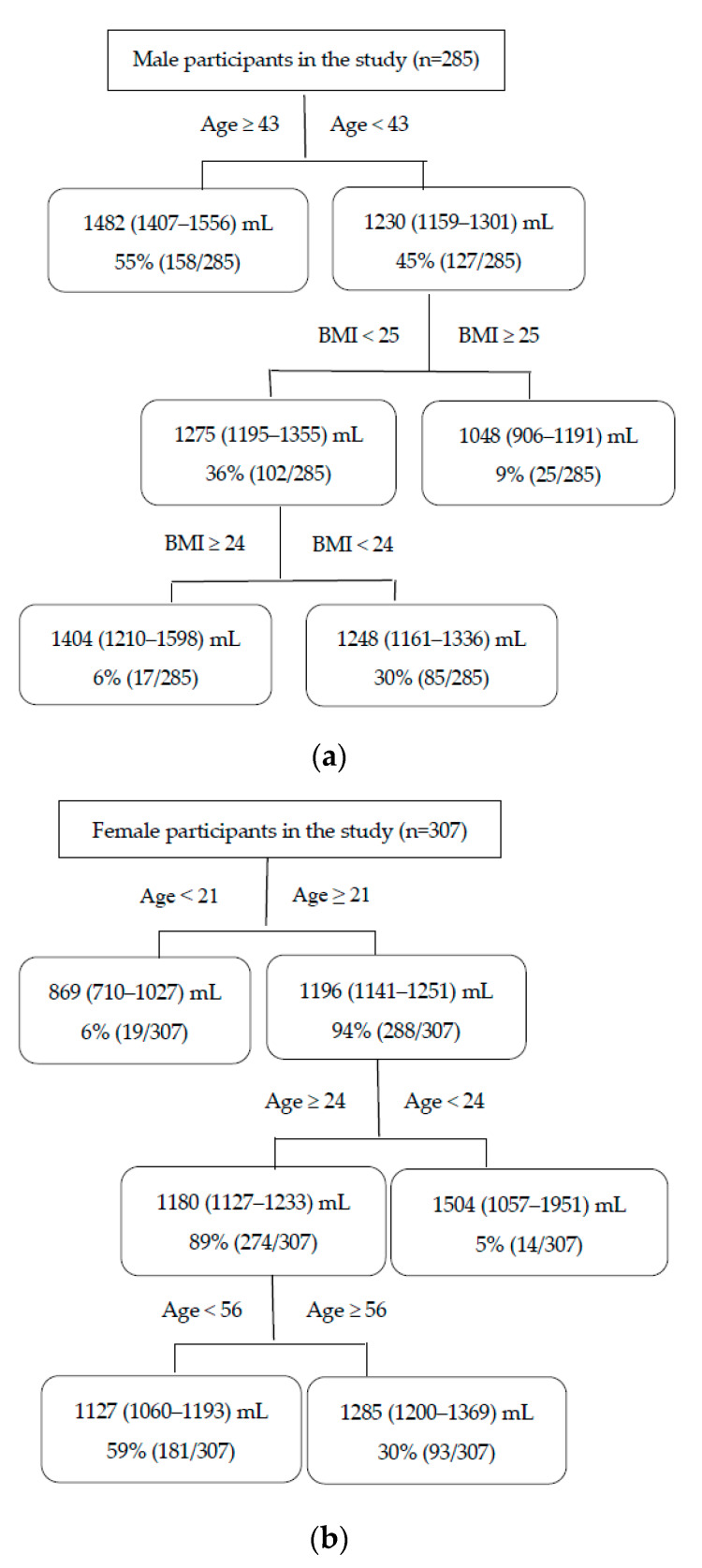
Impacts of age and BMI on daily water consumption among the adults in winter according to CART analysis in the study (age: years, BMI: kg/m^2^). The figures in the rectangle indicate the mean (95% CI) values of daily water consumption and the probability; (**a**): male, (**b**): female.

Figure 3 illustrates the impacts of age and BMI on daily water consumption among adults in winter. The group of adults consisted of 285 men and 307 women. The mean (95% CI) value of daily water consumption was 1370 (1316–1423) mL for men and 1176 (1123–1228) mL for women in winter.

According to Figure 3a, men aged ≥43 years (probability: 55%, mean (95% CI): 1482 (1407–1556) mL) consumed more water than those aged <43 years (probability: 45%, mean (95% CI): 1230 (1159–1301) mL). Furthermore, for men of age <43 years, the water ingestion was higher among individuals with a BMI of <25 kg/m^2^ (probability: 36%, mean (95% CI): 1275 (1195–1355) mL) than those with a BMI of ≥25 kg/m^2^ (probability: 9%, mean (95% CI): 1048 (906–1191) mL). Moreover, men aged <43 years with a BMI of <25 and ≥24 kg/m^2^ (probability: 6%, mean (95% CI): 1404 (1210–1598) mL) consumed more water than those with a BMI of <24 kg/m^2^ (probability: 30%, mean (95% CI): 1248 (1161–1336) mL).

For women in winter, age of 21 years was recognized as the first threshold: women aged ≥21 years (probability: 94%, mean (95% CI): 1196 (1141–1251) mL) had a higher water consumption than those aged <21 years (probability: 6%, mean (95% CI): 869 (710–1027) mL). Furthermore, women aged between ≥21 and 24 years (probability: 5%, mean (95% CI): 1504 (1057–1951) mL) consumed more water than those aged ≥24 years (probability: 89%, mean (95% CI): 1180 (1127–1233) mL). In addition, higher water consumption was detected among women aged ≥56 (probability: 30%, mean (95% CI): 1285 (1200–1369) mL) than those at aged from ≥24 to <56 years (probability: 59%, mean (95% CI): 1127 (1060–1193) mL) (Figure 3b).

### 3.5. Comparisons of the Results of Linear Regression and CART Analysis

In the regression model, gender, BMI, and location were confirmed as potential determinants associated with water consumption in summer, while gender and age were identified in winter. The results of CART analysis were consistent with regression analysis, in which BMI was the first classified factor in summer, and age was the leading factor in winter. Additionally, we determined that the thresholds of BMI were 23 kg/m^2^ for men and 18 kg/m^2^ for women in summer, respectively. In winter, the thresholds of age for men and women were 43-year old and 21-year old, respectively. 

## 4. Discussion

This study estimates the urban water consumption in an adult population in Wuxi, eastern China. According to the results, direct water consumption was the major source of total water consumption. The CART model suggested that BMI and age were the first classification factors, which were associated with water consumption in summer and winter, respectively. In summer, the threshold of BMI for men differed from women (BMI 23 vs. 18 kg/m^2^). The threshold of age for men was also different from women in winter (age 43 vs. 21 years)

Water ingestion rate is a crucial parameter that could be used to calculate the human exposure dose for assessing the exposure assessment of water pollutants. The median value (1351 mL/day) of water consumption in the study is close to the reported values of 1493 mL/day for Koreans [9] and 1466 mL/day for Americans [2]. The ratio of direct water to total water consumption (70.3%) is also similar to the findings in Korea (68.0%) [9]. We could not compare our results with those from the studies from Canada [6] and Ireland [13], where water consumption was defined differently. In addition, we found that only 15.5% of the adults had a water consumption of >2 L/day, which is employed as a default value for exposure assessment by the US Environmental Protection Agency and the World Health Organization. Hence, water ingestion of 2 L/day might be an overestimate for the adults in the study.

It is not surprising that the water consumption of the adults in summer was significantly higher than those in winter in the study. Seasonal variations in water consumption have been well- documented worldwide [9,37,38,39]. The seasonal effects are mainly attributed to the outdoor air temperature. Regnier, A. et al. [3] and Tani, Y. et al. [38] identified outdoor air temperature as the most significant factor for water consumption; this factor should be emphasized when assessing the amount of water that is consumed. In terms of gender difference, we found that men consumed more water than women, regardless of whether the water is direct or indirect. This finding can be explained by physiological differences between the two genders and is consistent with the results that were obtained in China [10,11,39], Ireland [13], and Korea [9]. However, a study from Sweden observed that women consumed more cold water than men [8], and Wright, C.J. et al. reported that women consumed more purchased water than men [40]. The differences may be explained by the different cultures and habits of the target populations.

According to the CART model, in summer, the BMI is the first identified factor for men (threshold: 23 kg/m^2^) and women (threshold: 18 kg/m^2^). In the multivariate analysis, we also found that a statistical association between water consumption and BMI in summer. Individuals with a high BMI may have a higher water demand than those with a low BMI. Similarly, an association between high BMI levels and high water intake was observed among the adults of China and the US [39,41]. However, a systematic review suggested a reducing effect on BMI of increased water consumption [42]. A survey that was conducted among Irish adults demonstrated an association between low fluid intake and high BMI [13]. These differences may reflect the regional characteristics of the study population. Due to the limitation of the study design, the causal relationship between BMI and water intake is worthy of further exploration.

The multivariate analysis demonstrated that water consumption increased with age in winter. Similarly, the CART analysis suggests that in winter, age is the first identified factor that may impact on water consumption, and the thresholds were 43 years for men and 21 years for women. According to a national survey, US adults over 21 years consumed the largest amount of water [2], whereas adults in the age range of 40–49 years had the highest water ingestion in Korea [9]. However, Roche, S.M. et al. [7] reported the highest tap water consumption among men aged 18–29 years, and subjects aged >60 years had a decreased tendency for tap water consumption in Canada. The thresholds of age for adults were identified using a machine-learning algorithm, thereby avoiding any bias that could be caused by artificial classification.

Our CART analysis demonstrates differences in water consumption among communities; men with a BMI of ≥23 kg/m^2^ who were residing Community A had a higher water consumption than those who were residing in Communities B and C in summer. The statistical differences of water consumption across the locations were also found in summer in the multivariate analysis. Community A had a higher socio-economic level than the other two communities, and the adults with high socio-economic backgrounds might pay more attention to water ingestion [30]. Therefore, this difference in water consumption among locations may be driven by socio-economic factors. Similarly, a study from the United Kingdom reported that lower-income adults may be at greater risk of inadequate hydration than higher-income adults [14]. Zhang et al. also observed that low socio-economic status may decrease fluid consumption, according to a national survey [10]. 

In the study, both of the CART and traditional regression models show that in different seasons, the leading factors associated with water consumption ware different, and they are the BMI in summer and the age in winter. However, the CART model appears to be more informative. For example, CART showed the thresholds for water consumption of the leading factors were different. These were a BMI of 23 kg/m^2^ for men and a BMI of 18 kg/m^2^ for women in summer and the age of 43 years old for men and the age of 21 years old for women in winter. These findings could help us identify the most important factors associated with water consumption and the subgroups that may have a high-level water consumption in the study site. For instance, in winter, men aged ≥43 years had a high-level value of water consumption (mean value: 1482 ml/day) in the study site, which means the group may have a high human exposure dose when the whole population is exposed to the same concentration of water pollutants. In the present study, we did not represent the water source patterns (e.g., purified water, reclaimed water) in the present study. This issue should be addressed in future studies that may better evaluate the association of water supply and demand [3,7,8].

To the best of our knowledge, this is the first study to estimate how demographic variables affect water consumption using the CART model. Both the high-order interactive effects and the thresholds of impact factors of water consumption have been identified in our study via CART analysis. The demographic variables were ranked according to their importance to water consumption. The findings will facilitate the targeting of subgroups of adults who have a high-level water intake rate by health risk assessors. Several limitations of the study should be addressed. First, several factors (e.., income, education level) were not taken into account in the present study. Second, the results of CART were prone to be changed by small differences in the dataset. Third, most of the participants were nonlabor workers with Han ethnicity, which suggests that it should be cautious when generalizing the findings to the whole population. Large-scale investigations will be required in the future.

## 5. Conclusions

Collectively, we estimated the urban water consumption among adults in Wuxi, China, and observed that the water consumption of the adults varied with gender, age, BMI, and location. 

The high-order interaction effects of variables that associated with water consumption are useful for accurately estimating water intake rates based on the characteristics of the study population and calculating a precise human exposure dose, which is an essential parameter for the exposure assessment of water pollutants. The findings may help risk assessors identify the high-risk subgroups for the exposure assessment of water pollutants.

## Figures and Tables

**Table 1:** Demographic characteristics of the participants in summer and winter.

Variables	Summer		Winter		Total	
	n	%	n	%	n	%
Total	596	100	592	100	1188	100
Gender						
Man	288	48.3	285	48.1	573	48.2
Woman	308	51.7	307	51.9	615	51.8
Age (years)						
18–34	198	33.2	198	33.4	396	33.3
35–54	193	32.4	192	32.4	385	32.4
≥55	205	34.4	202	34.1	407	34.3
Location (Community)						
A	198	33.2	202	34.1	400	33.7
B	200	33.6	197	33.3	397	33.4
C	198	33.2	193	32.6	391	32.9
Ethnicity						
Han	590	99.0	588	99.3	1178	99.2
Others	6	1.0	4	0.7	10	0.8
labor worker						
Yes	76	12.8	73	12.3	149	12.5
No	520	87.2	519	87.7	1039	87.5
BMI (kg/m^2^)						
<18.5	35	5.9	20	3.4	55	4.6
18.5–23.9	347	58.2	377	63.7	724	60.9
24–27.9	172	28.9	166	28.0	338	28.5
≥28	42	7.0	29	4.9	71	6.0

**Table 2:** Water consumption of the adults according to demographic variables in summer and winter.

Variables	n	Water Total (mL/day)		Direct Water (mL/day)		Indirect Water (mL/day)	
		Median	P25–P75	Median	P25–P75	Median	P25–P75
Summer	596	1525	1101–1970	1000	700–1400	471	343–612
Gender							
Man	288	1646	1203–2203	1100	750–1550	515	364–669
Woman	308	1442	1038–1825	960	600–1300	442	337–586
		*p* < 0.001		*p* < 0.001		*p* = 0.001	
Age (years)							
18–34	198	1323	978–1886	865	600–1300	424b	319–563
35–54	193	1511a	1136–2006	1075a	700–1400	463b	346–592
≥55	205	1638a	1237–2108	1100a	750–1500	542	394–689
		*p* < 0.001		*p* = 0.001		*p* < 0.001	
Location (Community)							
A	198	1531	1083–1902	950	673–1300	476	344–628
B	200	1640	1124–2107	1100	700–1500	504	359–621
C	198	1442	1097–1928	1000	650–1400	444	338–587
		*p* = 0.078		*p* = 0.048		*p* = 0.179	
BMI (kg/m^2^)							
<18.5	35	1074	840–1548	730	400–1100	381	284–471
18.5–23.9	347	1489c	1099–1915	1000c	680–1350	471c	344–620
24–27.9	172	1638c	1144–2190	1100c	700–1500	507c	344–612
≥28	42	1843c	1268–2371	1225c	750–1862	493	389–716
		*p* < 0.001		*p* < 0.001		*p* = 0.028	
Winter	592	1217	917–1555	850	600–1200	321	215–464
Gender							
Man	285	1321	1050–1644	1000	668–1300	364	220–474
Woman	307	1119	808–1458	800	550–1050	313	209–432
		*p* < 0.001		*p* < 0.001		*p* = 0.004	
Age (years)							
18–34	198	1116	773–1410	800	500–1100	275b	211–413
35–54	192	1231a	920–1617	915a	650–1250	315b	214–431
≥55	202	1315a	1058–1613	900a	650–1200	410	220–514
		*p* < 0.001		*p* = 0.003		*p* < 0.001	
Location (Community)							
A	202	1276	1014–1567	900	700–1100	342	216–468
B	197	1163	887–1516	800	550–1200	304	211–472
C	193	1172	861–1565	850	550–1200	325	217–455
		*p* = 0.085		*p* = 0.157		*p* = 0.767	
BMI (kg/m^2^)							
<18.5	20	1009	821–1450	800	425–850	270	210–463
18.5–23.9	377	1208d	906–1516	850	600–1200	316d	209–455
24–27.9	166	1320	1017–1680	900	700–1250	365	224–511
≥28	29	1016d	767–1414	800	425–975	274	214–418
		*p* = 0.003		*p* = 0.021		*p* = 0.025	

a: *p* < 0.05 (compared with age 18–34 years); b: *p* < 0.05 (compared with age ≥55 years); c: *p* < 0.05 (compared with BMI <18.5 kg/m^2^); d: *p* < 0.05 (compared with BMI 24–27.9 kg/m^2^).

**Table 3:** Impacts of demographic variables on the water consumption of the adults in summer and winter in multivariate analysis.

Variables	Coefficient	*p*-Value	95% (CI)
Summer (n = 596)			
Gender			
Woman	Referent		
Man	0.186	<0.001	0.099, 0.273
BMI	0.019	0.008	0.005, 0.034
Location (Community)			
A	Referent		
B	−0.113	0.034	−0.217, −0.008
C	−0.152	0.004	−0.255, −0.049
Constant	6.678	<0.001	6.343, 7.013
Winter (n = 592)			
Gender			
Woman	Referent		
Man	0.128	0.001	0.051, 0.204
Age	0.004	<0.001	0.002, 0.006
Location (Community)			
A	Referent		
B	−0.024	0.621	−0.118, 0.070
C	0.010	0.825	−0.083, 0.104
Constant	6.679	<0.001	6.541, 6.844

## Supplementary Materials

The following are available online at https://www.mdpi.com/1660-4601/17/9/2983/s1, Figure S1: Description of the multiple-stage random sampling method in the study. Table S1: Details of the questionnaire in the study. Table S2: Percentage of water content for the staple food in the study. Table S3: Water consumption of the adults for repeated measures in summer and winter.
