# Supplementary material for: Urban Water Consumption Patterns in an Adult Population in Wuxi, China: A Regression Tree Analysis
Source: Int J Environ Res Public Health. 2020 Apr 25;17(9):2983. doi: 10.3390/ijerph17092983 (PMC7246778; doi:10.3390/ijerph17092983)
Supplement: Supplementary file 1 [file ijerph-17-02983-s001.pdf]

International Journal of
Environmental Research and Public Health

MDPI

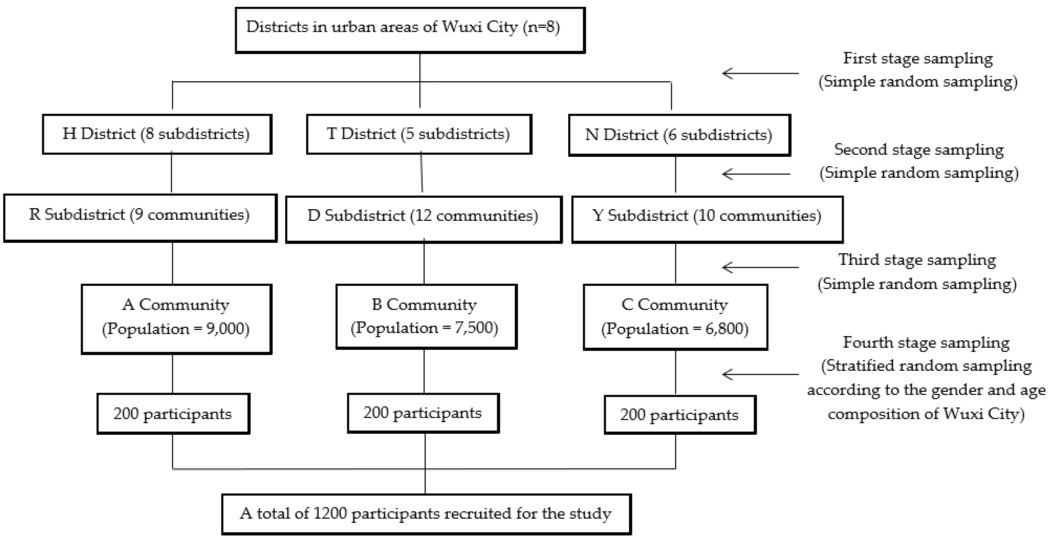

Supplementary Figure S1. Description of the multiple-stage random sampling method in the study.

Supplementary Table S1. Details of the questionnaire in the study.

Part 1 Basic information of the participants.

**1.**	**Code**
2.	Phone:
3.	Address:
4.	Date of birth
5.	Gender
6.	Ethnicity
7.	Labor worker 1. yes 2. no
8.	Height
9.	Weight

Part 2 Details of the self-reported diary.

1. Basic information.

**1.**	**Code**
2.	Phone
3.	Address
4.	Date of investigation (1) Begin: (2) End:

2.Content of diary.

**Time period**	**Meal**	**NO.**	**Contents**	**Volume (mL or gram)**
Morning	Before breakfast	1.	Water	
	Breakfast	2.	Water	
		3.	Soup	
		4.	Porridge	
		5.Staple food	1) Rice	
			2) Steamed bread	
			3) Noodle, rice noodle, and rice flour	
			4) Others	
	between breakfast and lunch	6.	Water	
Afternoon	Lunch	7.	Water	
		8.	Soup	
		9.	Porridge	
		10.Staple food	1) Rice	
			2) Steamed bread	
			3) Noodle, rice noodle, and rice flour	
			4) Others	
	between lunch and dinner	11.	Water	
Evening	Dinner	12.	Water	
		13.	Soup	
		14.	Porridge	
		15.staple food	1) Rice	
			2) Steamed bread	
			3) Noodle, rice noodle, and rice flour	
			4) Others	
	After dinner	16.	Water	
	Night	17.	Water	

Supplementary Table S2. Percentage of water content for the staple food in the study.

Food	Range of water content (%)	Median value (%) of water content used for calculation
Rice	70.6–71.1	70.9
Steamed bread	40.5–47.3	43.9
Noodle, rice noodle, rice flour and wonton	10.5–72.6	20.8
Others	10.5–72.6	20.8

Supplementary Table 3. Water consumption of the adults with repeated measures in summer and winter.

Variables	No. (%)	Median (Summer vs. Winter, mL/day)	Z	*p* value
Total	580 (100)	1,542 vs. 1,218	-10.943	<0.001
Gender				
Man	281 (48.4)	1,649 vs. 1,321	-7.862	<0.001
Woman	299 (51.6)	1,475 vs.1,119	-7.639	<0.001
Age (years)				
18–34	193 (33.3)	1,328 vs. 1,114	-5.910	<0.001
35–54	188 (32.4)	1,510 vs. 1,243	-5.883	<0.001
≥55	199 (34.3)	1,653 vs. 1,311	-7.105	<0.001
Location (Community)				
A	197 (44.0)	1,533 vs. 1,278	-4.871	<0.001
B	196 (33.8)	1,655 vs. 1,142	-7.632	<0.001
C	187 (32.2)	1,450 vs. 1,207	-6.308	<0.001
